# A National Pension Fund for Hospital Officials and Nurses

**Published:** 1887-05-14

**Authors:** 


### A National Pension Fund for Hospital Officials and Nurses.

The Views of Officials, Sisters, Nurses, and others.

From the Matron of the Beckett Hospital, Barnsley.

I gladly send in my name as a subscriber to the National Pension Fund for Nurses. I am greatly interested in the scheme. I hope all nurses would be eligible as members, whether they are hospital, private, or district nurses, providing they are hospital-trained nurses. I should like to say that if the Fund could be established and worked on a similar basis to the Friendly Societies' Sick Clubs—that is, a weekly sum to be paid to nurses who are unable to work through sickness, and a fixed sum to be paid to the relations in case of death—I think if something of this sort were to be carried out, nearly all nurses would gladly become subscribers to the Fund. My four nurses will send in their names later on.

By One of the Nurses at the Henrietta Street Nursing Institute.

Having seen in The Hospital that you were trying to get up an Annuity Fund for trained nurses, I would like the names of those that wished it. I gladly send mine, for I have thought for a long time it was a thing greatly needed. I am sure, when nurses come to know of it, they will appreciate it very much. I know of many also myself that would gladly subscribe to it yearly, knowing we were sure of something when sick and our health gave way, and we could no longer follow our noble profession. I think such a good thing ought to be taken up by royalty and the nobility, as nurses ought to be thought of as well as hospitals, who sacrifice their lives and health for the good of their fellow-creatures. Wishing you every success in your noble undertaking.

From the Matron of the Victoria Hospital for Burnley and District.

It is with great pleasure I add my testimony to the great need existing for a Nurses' Pension Fund. Having been practically engaged in the profession for over nine years, I have frequently heard nurses express a wish that something of the kind could be formulated. I am sure there will be no lack of subscribers, if the Fund is once established, and the nurses' attention drawn to it. A hospital nurse's work so very often engrosses her mind to the exclusion of what is being done in the outer world, that no doubt there are many who have not yet heard of the project, or you would have had more names sent. It would also be of very great advantage if in connection with, or apart from the above, as may be deemed most advisable, there could be formed a Convalescent Fund from which nurses could draw a certain sum weekly, say after a severe illness, or when tired and needing a rest. I have known good nurses whose health has broken, and who have had to give up work entirely, when a little timely rest and change of scene (which they possibly could not afford at the time) would have enabled them to continue useful for years. My seven nurses wish me to forward their names, and express their sincere appreciation of the scheme. I also add my own and the secretary's.

